# Metagenomic and Metabolomic Correlates of Immunotherapy Response in Non-Small Cell Lung Cancer

**DOI:** 10.21203/rs.3.rs-10107631/v1

**Published:** 2026-07-09

**Authors:** Acadia W. Buro, Maria F. Gomez, Yumi Kim, Nathan P. Ward, Mark Umbarger, Lanying Ma, Ayin Vala, Stephanie Hogue, Wildson Viera Silva, Alexis Bailey, Christine M. Pierce, Youngchul Kim, Gina M. DeNicola, Doratha A. Byrd, Lary A. Robinson

**Affiliations:** Moffitt Cancer Center; Moffitt Cancer Center; Moffitt Cancer Center; Moffitt Cancer Center; Sail Biomedicines; PragmaBio; PragmaBio; Moffitt Cancer Center; Moffitt Cancer Center; Moffitt Cancer Center; Moffitt Cancer Center; Moffitt Cancer Center; MRL, Merck & Co., Inc; Moffitt Cancer Center; Moffitt Cancer Center

**Keywords:** Immunotherapy, Gut microbiome, Non-small Cell Lung Cancer, Metabolomics, Immune Checkpoint Inhibitors

## Abstract

**Background:**

The gut microbiome may influence cancer treatment response, perhaps by immune system interactions, but studies are limited among non-small cell lung cancer (NSCLC) patients. We investigated associations of the pre-treatment gut microbiome and serum metabolome/lipidome with immune checkpoint inhibitor (ICI) response among patients with stage III-IV NSCLC.

**Methods:**

We conducted an observational cohort study with fecal and blood collection among 66 patients with stage III-IV NSCLC undergoing ICI therapy, using an updated definition of clinical benefit. Fecal whole genome sequencing, plasma untargeted metabolomics, and serum lipidomics were conducted using liquid chromatography mass spectrometry. Multivariable logistic regression estimated associations of alpha/beta diversity, microbial abundance, metabolites, and lipids with clinical benefit. Microbial taxa, metabolites, lipids, and significant lipids correlations were examined.

**Results:**

Microbiome composition (beta diversity) differed between participants with and without clinical benefit (*P* = 0.03). Those with higher relative abundance of *Bifidobacterium* were less likely (OR per 1-SD = 0.51, 95%CI = 0.25–0.92, *P* = 0.04) to have clinical benefit. Those with higher *Ruminococcus* prevalence were more likely (OR = 7.00, 95%CI = 1.80–34.47, *P* = 0.01) to have clinical benefit. Clinical benefit participants had higher serum concentration of 4-Imidazoleacetate (OR = 6.34, 95%CI = 2.36–22.29, *P* = 0.001), 6-Bromotryptophan (OR = 3.84, 95%CI = 1.80–10.17, *P* = 0.002), and lyso-phosphatidylcholines (OR = 4.52, 95%CI = 1.59–17.19, *P* = 0.01) compared to no clinical benefit, though these findings were not statistically significant after multiple corrections.

**Conclusions:**

This hypothesis-generating study found *Ruminococcus* was positively, and *Bifidobacterium* inversely, associated with ICI response among NSCLC patients. The gut microbiome and related metabolites/lipids were found to be associated with ICI clinical benefit among NSCLC patients. Larger, diverse longitudinal studies are needed to clarify the associations of the microbiome and related metabolites with ICI response among NSCLC patients.

## BACKGROUND

Immune checkpoint inhibitor (ICIs) therapy among non-small-cell lung cancer (NSCLC) patients began in March, 2015 with the first FDA-approved use of nivolumab targeting PD-1 for treatment of metastatic NSCLC after chemotherapy failure,^1^ and subsequently in May, 2020 in combination with ipilimumab targeting CTLA-4 along with chemotherapy for first-line treatment of metastatic NSCLC.^2^ Often there were dramatic results, but only 20% of patients achieved a clinical response and 8% survived long-term with slightly improved results with more toxic combination therapy.^3^ Recent evidence suggests that the gut microbiome plays a key role in the response to immunotherapies, with multiple genera and species having been associated with a favorable response.^4–10^ Importantly, the gut microbiome is potentially modifiable, offering opportunity to intervene to change the microbiome and improve patient outcomes. Despite exciting preliminary evidence, results across studies have been inconsistent. In fact, a detailed approach is critically needed to define measures of the gut microbiome at the species level and measurements of products regulated and produced by the gut microbiome, such as metabolites and lipids.^11^

Additionally, more appropriate clinical definitions of ICI response should be used. When immunotherapy was first introduced, response was measured with the same methodology as for chemotherapy using conventional RECIST v1.1 criteria.^12^ This focuses on radiographic unidimensional measurement of the target tumor size with a size decrease considered to be a partial response (PR) and complete tumor resolution (CR) considered indicators of ICI effectiveness, while stable tumor size (SD) or tumor mass enlargement are considered progressive disease (PD) indicating resistance of the tumor to ICIs—the so-called 2 vs. 2 response.^13^ However, we have subsequently learned that some tumors responding to ICI treatment have a transient increase in tumor size with the influx of T-cells and other inflammatory cells plus edema with tumor necrosis. This “pseudo-progression” may lead to misdiagnosis of no clinical benefit (appearing to have progressive or stable disease) when in fact the tumor shrinks in the following months.^13–16^

Therefore, we propose that a more reliable clinical indicator of ICI response is the “clinical benefit,” which includes best response of CR, PR and SD seen within 12 months, while PD within 12 months indicates no clinical benefit (i.e., 3 vs. 1 response). Therefore, a lack of cancer progression at the 12-month mark indicates a definite treatment response, despite the appearance of stable disease. In the context of immunotherapy treatment for aggressive lung cancer, this “clinical benefit” is termed best response within 12 months of CR/PR/SD and/or experienced progression-free survival (PFS) for at least 365 days. Although the response may not be durable for subsequent years and may not result in improved long-term survival, there was a definite *initial* response to the ICIs.

Recent studies in humans with various cancer types (e.g., melanoma and lung cancer) have demonstrated that the gut microbiome plays a role in influencing the response to anticancer therapy, although the mechanisms remain unclear.^4–10^ Since considerable evidence exists to support a role for gut microbiome in modulating the response to immunotherapy in other tumors, we postulated that response to immunotherapy among NSCLC patients was also influenced by the gut microbiome and the metabolic effects of specific microbes. To better understand the role of the gut microbiome in ICI response and potential mechanisms involved, we prospectively studied the associations of the pretreatment gut microbiome and serum metabolome/lipidome with “clinical benefit” to ICI(i.e., PFS at 12 months) among ICI-treatment-naive patients with stage III-IVNSCLC.

## METHODS

### Study Population

Between June, 2016 and February, 2019, this prospective, observational study cohort recruited adult patients with stage III-IV NSCLC patients (TMN 8th Edition) with squamous cell carcinoma, adenocarcinoma or mixed NSCLC subtypes who were scheduled to undergo ICI therapy at Moffitt Cancer Center as a single agent or in combination with other therapies with most having failed prior chemotherapy. The very few stage III patients had failed chemoradiotherapy prior to starting ICI therapy. Early morning pre-treatment stool and blood samples were obtained for analysis. Informed consent was obtained from all patients. This study was approved by Advarra IRB, MCC# 18611, PRO00017235.

### Clinical Metadata Abstraction

Clinical information about socio-demographics were abstracted from the Moffitt Cancer Center Electronic Patient Questionnaire and medical record. Prior to receiving treatment, patients’ medical records were used to collect information on the diagnosis (histology and stage), number/types of previous treatments received, current medications, recent use of antimicrobial therapy, PD-L1 measured by immunohistochemistry staining (IHC 22C3 pharmDx) and CTLA-4 measured by ELISA (enzyme-linked immunosorbent assay) status, height/weight, ECOG-PS (Eastern Cooperative Oncology Group Performance Status), and co-morbidities. Medical records were reviewed monthly until clinician-confirmed tumor progression or death. Immunotherapeutic response, PFS, and treatment-related adverse events at each clinical visit were recorded.

A single radiologist evaluated all patients’ radiological images to assess treatment response using RECIST v1.1 criteria to minimize variability by clinician or whether patients received treatment through a clinical trial or standard of care. Although a newer response criteria for randomized immunotherapy treatment trials was proposed in 2018, iRECIST, this methodology was not felt to be as useful for this observational study where we wanted a more comparable and simpler endpoint for ICI response, and specifically clinical benefit.^17^ Therefore for this study, we defined patients experiencing “clinical benefit,” as a best response of CR/PR/SD within 12 months and/or PFS time of ≥ 12 months, where PFS was calculated as time from ICI start date-to-date of progression, death, or last contact.

### Sample Collection

#### Fecal Samples

Each patient was given a custom at-home stool collection kit, optimized for microbiome studies conducted among cancer patients, and was asked to collect the samples from their early/mid-morning bowel movement no sooner than 24–72 hours prior to their first immunotherapy treatment. Detailed collection instructions have been described elsewhere.^18^ Each patient stored the tubes and test card inside the cardboard box and kept it indoors at room temperature until their first immunotherapy infusion. Participants provided the time/date at which specimens were collected. They also characterized the stool according to one of seven types on the Bristol stool chart^19^ and provided dietary information at the time stool was collected.

The Research Coordinator retrieved the fecal specimens from patients in clinic or via courier delivery. The Hemoccult II SENSA card (FOBT) was individually stored inside a Ziploc bag, placed vertically inside a box, and stored at −80°C until prepared for extraction, when one frozen card slide window per participant was cut using a sterile, disposable scalpel and transferred to a sterile microcentrifuge tube. Of note, several sampling controls (i.e., empty FOBT cards and extraction reagents) were also processed alongside participant samples to account for potential contaminants in the cards. DNA extraction was then performed using a modified version of the Qiagen^®^ Power Soil DNA Extraction Kit protocol (Qiagen, Hilden, Germany), courtesy of Dr. Nicholas Chia.^20,21^ Briefly, to ensure full lysis of the FOBT card matrix, the lytic step was modified to where the card slide window was lysed using the Precellys24 homogenizer (Bertin Instruments, Montigny-le-Bretonneux, France) at 7400 rpm for one minute. Centrifugation cycles, according to the kit’s protocol, were only to be 30 seconds but were increased to one minute. Incubation steps that were originally set at 5 minutes were increased to 30 minutes. DNA was quantified using Qubit and quality ascertained via Nanodrop readings. The resultant~100uL of DNA was divided into 2, 50uL aliquots that were then frozen at −80°C until sequencing.

#### Blood Samples:

Blood was collected from each participant prior to their first treatment. Pre-treatment, 10 mL of blood was collected in one red-topped vacutainer tube that sat for 20–60 minutes at room temperature before being centrifuged at 1500–1800g for 20 minutes at room temperature to separate serum from clotting factors. Up to five 1 mL serum aliquots were generated and snap frozen in liquid nitrogen (LN) before being stored at −80°C. Two, lavender-topped EDTA vacutainer tubes were also used to collect 10 mL of blood each: one was frozen at −80°C without any processing, while the other tube was centrifuged at 1500–1800 g at 4°C for 15 minutes to isolate five, 1mL aliquots of plasma. After snap freezing in LN, these aliquots were stored at −80°C. Frozen serum specimens underwent lipidomics analysis by the Moffitt study team and frozen plasma samples were sent to Senda Biosciences (Cambridge, MA) for targeted and untargeted metabolomic analysis.

### Fecal Sample Whole Genome Sequencing

One frozen, 50uL aliquot of DNA was sent to SeqMatic (Freemont, CA) for whole metagenome shotgun sequencing on the Ilumina NovaSeq 6000 system. Paired-end 2×150 chemistry: each sample received dual unique indexes and samples were multiplexed on an S4 flowcell, producing approximately 40M reads per sample. Reads were then quality filtered, after which de-novo assembly using MegaHIT was performed. Known positive control aliquots were used including in the whole genome sequencing to ensure validity of processes. Data generated from this study were used to identify and quantitate bacteria present within feces.

### Plasma Metabolite Analysis

Metabolites in plasma were quantified by Senda Biosciences (Cambridge, MA). Untargeted global metabolomics was performed on plasma samples from all donors via ultra-high performance liquid chromatography/tandem mass spectroscopy methods, including: (1) acidic positive ion conditions, one chromatographically optimized for more hydrophilic compounds, the other chromatographically optimized for more hydrophobic compounds; (2) basic negative ion optimized conditions; and (3) negative ionization. In total, 1065 compounds were detected in the plasma samples. Raw signals were median normalized and missing values were imputed using the half-minimum detected value for that compound.

### Serum Lipidome Analysis

Serum samples were extracted with chloroform:methanol (v:v = 1:2) extraction solvent containing internal standards at the following concentrations: 5nM D7-Sphinganine (Avanti Polar Lipids Inc., Cat# 860658), 12.5nM D3-Deoxysphinganine (Avanti Polar Lipids Inc., Cat# 860474), 5nM N-12:0–1-deoxysphinganine (Avanti Polar Lipids Inc., Cat# 860481), and 5nM N-C12-deoxysphingosine (Avanti Polar Lipids Inc., Cat# 860455). 50 μL of serum was extracted with 450 uL of lipid extraction solvent in ice-cold water using the Biorupter^™^ UCD-200 sonicator for 5 min (30sec sonication and 30sec rest cycle; high voltage mode). The lipid extracts were cleared by centrifugation (17,000g, 20°C, 10min), and the lipids in the supernatant were analyzed by LC-MS/MS as previously described.^22^ In brief, chromatographic separation was conducted on a Brownlee SPP C18 column (2.1mm × 75mm, 2.7μm particle size, Perkin Elmer, Waltham, MA) using mobile phase A (100% H_2_O containing 0.1% formic acid and 1% of 1M NH_4_OAc) and B (1:1 acetonitrile:isopropanol containing 0.1% formic acid and 1% of 1M NH_4_OAc). The gradient was programmed as follows: 0–2 min 35% B, 2–8 min from 35 to 80% B, 8–22 min from 80 to 99% B, 22–36 min 99% B, and 36.1–40 min from 99 to 35% B. The flow rate was 0.400 mL/min. The HPLC was coupled with a Q Exactive HF mass spectrometer equipped with HESI (Thermo Fisher Scientific, Waltham, MA), which was operated as follows: MS^2^ scan conditions were applied in positive mode, the scan range was from m/z 120–1000, and the resolution was 120,000 for MS, and 30,000 for DDMS^2^ (top 10), and the AGC target was 3e^6^ for full MS and 1e^5^ for DDMS^2^, allowing ions to accumulate for up to 200ms for full MS and 50ms for DDMS^2^. For the MS/MS scan the following conditions were used: NCE at 20, 30, and 40a.u., minimum AGC 5e^2^, and dynamic exclusion of previously sampled peaks for 8sec.

QC lipid samples were included to check the technical variability and were prepared by pooling an equal volume of lipid extract from all serum samples. QC samples were included after every 10th analytic sample and monitored for changes in peak area, width, and retention time to determine the performance of the LC-MS/MS analysis. The lipid peaks were identified, aligned, and exported using MS-DIAL.^23 21 22^ The data were further normalized to the median value of total lipid signals. Only lipids fully identified by MS^2^ spectra were included in the analysis.

### Statistical Analysis

Participant characteristics were summarized using descriptive statistics, and Kruskal–Wallis tests were used to compare participants with and without clinical benefit. The outcome was coded dichotomously as clinical benefit vs. no clinical benefit, where clinical benefit was defined as best response of CR/PR/SD within 12 months and/or PFS time of ≥ 12months.

To investigate the associations of microbial richness and evenness with immunotherapy clinical benefit, we estimated Observed species and Shannon index alpha diversity metrics based on the average of 10 rarefaction samplings. Metrics were modeled continuously in the analyses. Standardized metrics for beta diversity were created using the Bray-Curtis distance matrix and visually assessed via PCoA plots. Beta diversity was further analyzed using the microbiome regression-based kernel association test (MiRKAT) permutation method to estimate a covariate-adjusted p-value for compositional differences by clinical benefit based on the Bray-Curtis dissimilarity matrices.

To further evaluate the contribution of clinical and treatment-related covariates to overall microbiome composition, we conducted Permutational Multivariate Analysis of Variance (PERMANOVA) using the adonis function in the vegan package in R. The percentage of variability (R^2^) explained by sex, age, body mass index (BMI), prior treatment, antibiotic use, proton pump inhibitor (PPI) use, and probiotic use within one year prior to sample collection was estimated based on Bray-Curtis dissimilarity matrices.

Relative abundance transformed at the center-log ratio(CLR) and reported per one standard deviation (1-SD), in addition to prevalence (presence/absence), were estimated at the genus and species taxonomical levels, with specific attention given to *a priori*-selected bacteria based on prior literature. At the genus level, we included *Ruminococcus*, *Akkermansia*, *Blautia*, *Faecalibacterium*, *Bacteroides*, *Escherichia*, and *Bifidobacterium*,^24,25^ and at the species level, we included *Akkermansia muciniphila*,^*8*^*Alistipes putredinis*, *Bifidobacterium longum*, and *Prevotella copri*.^26^ Exploratory relative abundance and prevalence analysis was conducted for genera and species identified in at least half of the samples with an average abundance across samples of ≥ 0.1%. *Bacteroides* and *Blautia* were excluded from the presence analysis due to their presence in all samples.

In the metabolomics analyses, the abundance of features in each sample (n_patients_=62) was normalized by volume and scaled to have a median of one, and missing values were imputed with the minimum detected value.^24^ The abundance of lipids in each sample (n_patients_=35) was calculated as peak intensity and normalized to account for sampling variability.

Multivariable logistic regression models were used to estimate associations of the microbiome features, metabolites, and lipids with clinical benefit. We visualized the strongest correlations between metabolites and lipids with microbiome species using heat maps of the partial Spearman correlation coefficients. Rows and columns were hierarchically clustered using Wald’s methods with Euclidean distance as the dissimilarity metric. We included dendrograms to display clustering patterns among metabolites/lipids and microbial species, with cluster boundaries predefined into 6 clusters for microbiome and 5 clusters for metabolites/lipids. The clusters represent groups of features with similar correlation patterns based on the partial Spearman correlations.

The following covariates were included in all models based on relevance, prior literature, biological plausibility, and feature selection to avoid overfitting: biological sex, age at diagnosis, Bristol stool sample type, proton-pump inhibitors or other gastrointestinal medication prior to treatment, antibiotics within 12 months prior to treatment, current probiotic/prebiotic use, prior thoracic radiation, chemotherapy, and targeted treatment. Analyses were conducted with R version 4.4.2 using an alpha level of 0.05 for *a priori* analyses. All p-values for exploratory tests were adjusted for type one error inflation using a Bonferroni adjustment for multiple comparison correction.

Sensitivity analyses were conducted using stratified multivariable logistic regression models to evaluate whether associations between a priori selected bacterial taxa and clinical benefit differed by antibiotic use (yes vs. no), probiotic use (non-users only), and PPI use (users only). Analyses were restricted to strata with sufficient sample size to ensure stable parameter estimation. Models included the same covariates as the primary analyses.

## RESULTS

### Participant Characteristics

**Figure 1:**
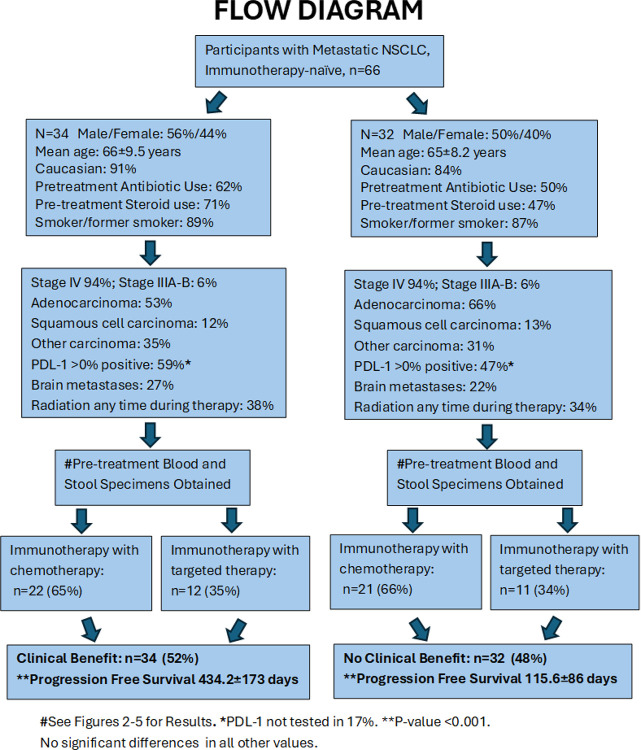
CONSORT Style Flow Diagram of Patient Demographics and Clinical Results.

Table 1 summarizes participant characteristics. Participants in the clinical benefit group (N = 34) had a mean age of 66.0 years and were 50% female and 91.2% white. 61.8% had a history of antibiotic use within a year from treatment start. There were no statistically significant differences in participant characteristics including pre-treatment steroid use and brain metastases by clinical benefit except for PFS in day (p < 0.001). The number of patients with a positive PDL-1 was not significantly different (46.9% no clinical benefit versus 58.8% clinical benefit, p = 0.33). In the PDL-1 positive tumors, there was no difference in the mean numerical % result. Although there was numerically higher steroid use among those with clinical benefit vs. those without (70.6% vs. 46.9%), this finding was not statistically significant. Virtually all patients in both groups had concurrent therapy with ICI plus chemotherapy or targeted therapy, either started together or more commonly added months later depending on the patient response and the preference of the treating oncologist. A CONSORT style flow chart (Fig. 1) graphically displays these results.

### Associations of the Gut Microbiome with Clinical Benefit to ICI

**Figure 2:**
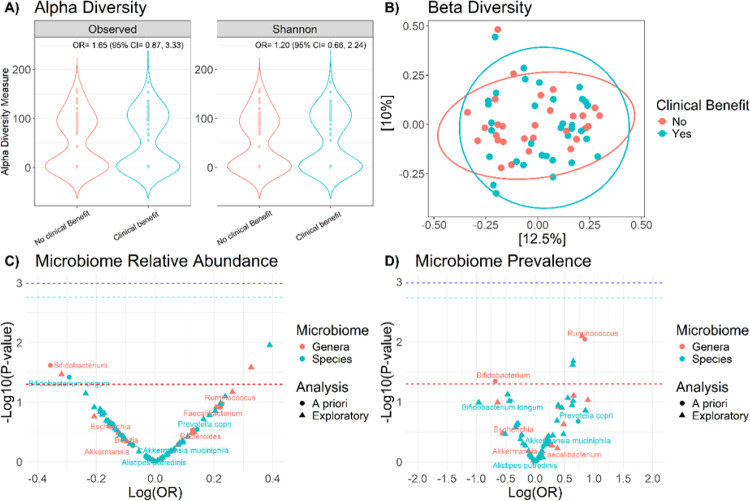
Associations of microbiome features with clinical benefit (benefit n=32 vs. no benefit n=34) in a cohort of patients diagnosed with non-small cell lung cancer at Moffitt Cancer, N=66. (A) Violin plots of the untransformed alpha diversity distribution among individuals with and without clinical benefit. Odd ratios (OR) and 95% confidence intervals of clinical benefit (no benefit as the reference) were estimated using logistic regression models of the z-scored alpha diversity metrics. (B) Principal Coordinate Analysis (PCoA) of beta diversity based on the Bray-Curtis distance matrix by clinical benefit. (C) Volcano plot of *a priori* and exploratory microbial species and genera relative abundance. Odd ratios (OR) and 95% confidence intervals of clinical benefit (no benefit as the reference) were estimated using logistic regression models of the center-log ratio and z-scored transformed relative abundances. Statistical significance for *a priori* bacteria is shown by the red dotted line (alpha level 0.05), exploratory species is shown in the blue dotted line (alpha level of 0.001), and genera is shown in the turquoise dotted line (alpha level 0.002). (D) Volcano plot of *a priori* and exploratory microbial species and genera prevalence (presence/absence). Odd ratios (OR) and 95% confidence intervals of clinical benefit (no benefit as the reference) were estimated using logistic regression models of the presence (relative abundance >0) or absence (relative abundance = 0) of each bacteria taxa. Statistical significance for *a priori* bacteria is shown by the red dotted line (alpha level 0.05), exploratory species is shown in the blue dotted line (alpha level of 0.001), and genera is shown in the turquoise dotted line (alpha level 0.002). Logistic regression models were adjusted for sex, age at diagnosis, BMI (kg/m^2^), additional treatment (chemotherapy, radiation, or targeted therapy), Bristol Stool type, antibiotic use 12 months prior to collection, PPI use, and probiotic use.

Alpha diversity was not statistically significantly associated with clinical benefit, as shown in Figure 2A (**Supplementary Table 1**). However, observed species and Shannon were generally positively associated with higher odds of clinical benefit (Observed, OR=1.65, 95% CI=0.87–3.33 and Shannon OR=1.20, 95% CI=0.66–2.24). There were no overall differences in the composition of gut microbiome of individuals with clinical benefit compared to those without assessed by PCoA based on Bray-Curtis distance (Figure 2B; **Supplementary Table 1**); however, MiRKAT test indicated that gut microbiome composition based on Bray-Curtis distances statistically significantly differed between those with clinical benefit and those without (p=0.03) (**Supplementary Table 2**). PERMANOVA analyses demonstrated that sex explained a small but statistically significant proportion of overall microbiome compositional variation (R^2^=2.8%, p=0.02), while age, BMI, prior treatment, antibiotic use, PPI use, and probiotic use each explained small and non-significant proportions of the variation (**Supplementary Figure 1**). Consistent with these findings, no significant differences in overall microbiome composition based on antibiotic, PPI, or probiotic use were observed using Bray-Curtis distances. However, the number of participants reporting probiotic use and those not using PPI was limited.

Multiple bacteria were associated with clinical benefit. In *a priori* analyses shown in Figure 2C (**Supplementary Table 1**), considered per 1-SD increase in the CLR-transformed relative abundances of species *Bifidobacterium longum* and genus *Bifidobacterium*, there were 49% and 56% lower odds of clinical benefit (95% CI=0.25–0.92, *P*=0.04; and 95% CI=0.20–0.84, *P*=0.02), respectively. The presence of genus *Ruminococcus* was strongly positively associated with the odds of clinical benefit. For example, there was 7-fold higher odds of clinical benefit in those with the presence of *Ruminococcus*(OR=7.00, 95% CI 1.80–34.47, *P*=0.01) compared to those without it (Figure 2D). The associations of exploratory-selected taxa relative abundances and presences were not statistically significant after correction for multiple testing.

In stratified analyses (**Supplementary Figure 2**), associations between a priori bacterial taxa and clinical benefit were generally consistent across subgroups but varied in magnitude. Among participants without antibiotic use, a one-standard deviation increase in the relative abundance of *Bifidobacterium* was associated with a non-significant increase in the odds of clinical benefit. For *Bifidobacterium longum*, associations appeared to differ by probiotic and PPI use. Most participants reported no probiotic use (72%; n=57) and PPI use (n=52), and stratified analyses demonstrated inverse associations within both subgroups. The small number of participants reporting probiotic use but not PPI use precluded further subgroup evaluation.

#### Metabolites, Lipids, and Clinical Benefit

**Figure 3:**
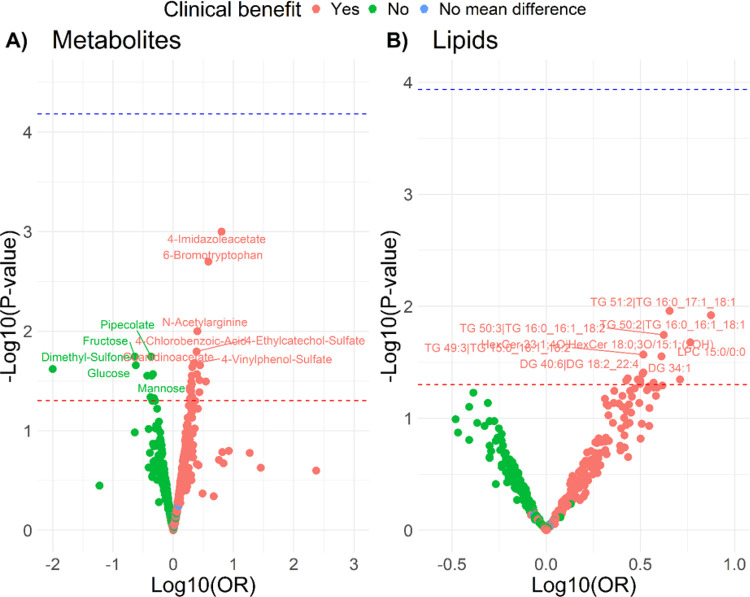
Associations of metabolites (62 patients; 762 metabolites) and lipids (36 patients; 430 metabolites) with clinical benefit in a cohort of patients diagnosed with non-small cell lung cancer at Moffitt Cancer. Volcano plots of the log10 odds ratios by the −log10 of the unadjusted *P-values* of the association of (A) metabolites and (B) lipids with clinical benefit (n=30, n=21, respectively) compared to those without a clinical benefit (n=32, n=15, respectively). Odd ratios (OR) and 95% confidence intervals of clinical benefit (no benefit as the reference) were estimated using logistic regression models of the z-scored metabolites and lipids. The colors represented whether higher mean levels of metabolites and lipids were observed among those with a clinical benefit (red=Yes), those without a clinical benefit (green=No), and no difference in the mean levels (blue=No difference). Statistical significance at an alpha level of 0.05 is shown by the red dotted line (alpha level 0.05 for both graphs). Statistical significance after correction for multiple testing using the Bonferroni method is shown in the blue dotted line (α=6.61e-06 for metabolites and α=0.0001 for lipids). Logistic regression models were adjusted for sex, age at diagnosis, BMI (kg/m^2^), additional treatment (chemotherapy, radiation, or targeted therapy), Bristol Stool type, antibiotic use 12 months prior to collection, PPI use, and probiotic use.

Considering a per 1-SD increase in the normalized metabolites and lipids, there were no statistically significant associations after correction for multiple testing observed in the associations of metabolomics (Figure 3A) and lipids (Figure 3B) with clinical benefit (see **Supplemental Tables 3 and 4** for exact estimates).

However, metabolites involved in histamine and tryptophan metabolism (e.g., 4-Imidazolacetate OR=6.34, 95% CI=2.36–22.29; *P*=0.001; and 6-Bromotryptophan OR=3.48, 95% CI=1.80–10.17, *P*=0.001) were associated with higher odds of clinical benefit. This was also the case for some triacylglycerols involved in lipid signaling and immune response activation (e.g., TG 51:2|TG 16:0_17:1_18:1: OR=4.52, 95% CI=1.59–17.19, *P*=0.01 and TG 50:2|TG 16:0_16:1_18:1: OR=7.49, 95% CI=1.88–46.48, *P*=0.01).

#### Metabolites, Lipids, and the Gut Microbiome

**Figure 4:**
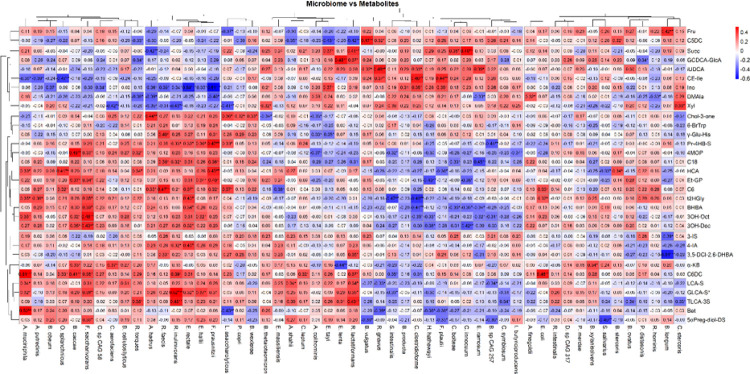
Strongest associations of microbial species with circulating metabolites among patients diagnosed with NSCLC, N=62. Coefficients of partial Spearman for the strongest correlation tests between CLR z-score transformed microbiome relative abundance at the species level with normalized and z-scored transformed circulating metabolites. Microbiome and metabolites were hierarchically clustered using Wald’s method with Euclidean distances, with predefined clusters of six for microbiome and five for metabolites. Models were adjusted for sex, age at diagnosis, BMI (kg/m^2^), additional treatment (chemotherapy, radiation, or targeted therapy), antibiotic use 12 months prior to collection, PPI use, and probiotic use. Strongest correlation and p-values are presented at * 0.05, ** 0.01, *** <0.001. The significance after the Bonferroni correction for multiple testing is 1.14 × 10^ (− 6). Abbreviations in **Supplementary Table 7**.

**Figure 5:**
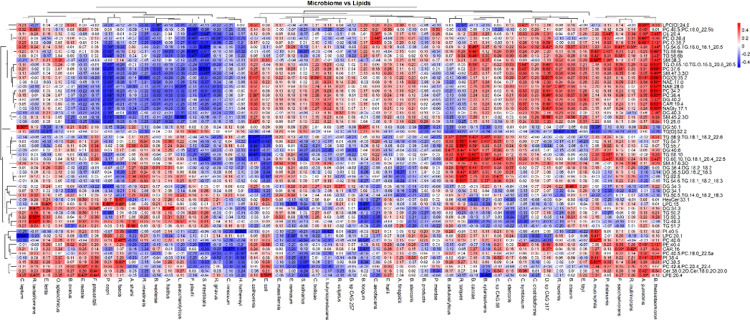
Strongest associations of microbial species with circulating lipidomics among patients diagnosed with NSCLC, N=35. Coefficients of partial Spearman for the strongest correlation tests between CLR z-score transformed microbiome relative abundance at the species level with normalized and z-scored transformed circulating lipidomics. Models were adjusted for sex, age at diagnosis, and BMI (kg/m^2^). Strongest correlation and p-values are presented at * 0.05, ** 0.01, *** <0.001. The significance after the Bonferroni correction for multiple testing is 2.19 × 10^ (− 6). Abbreviations in **Supplementary Table 7**.

Correlations among metabolites (Figure 4), lipids (Figure 5), and the gut microbiome were moderate, but generally not statistically significant after Bonferroni correction (**Supplementary Tables 5 and 6; Abbreviations Supplementary Table 7**).

Several associations were observed between the species *Bifidobacterium longum*, metabolites, and lipids. Notably, fructose was positively correlated with the species *Bifidobacterium longum*(R^2^= 0.42; *P*<0.001), while 3–5 dichloro-2–6 dihydroxy benzoic acid and 3-indoxyl sulfate were inversely associated with *Bifidobacterium longum*(R^2^=−0.54, P <0.001 and R^2^=−0.39, *P* <0.001, respectively). *Bifidobacterium longum* was also inversely correlated with carnitine-related metabolites(Palmitoyl carnitine/CAR 16:0, R^2^=−0.37, *P*=0.04) and pro-inflammatory diacylglycerols(DG 34:3|DG 16:1_18:2, R^2^=−0.40, *P*= 0.02); and was positively correlated with long-chain monounsaturated fatty acids(DG 50:1, R^2^=0.41, *P*=0.02) involved in membrane and lipid storage.

## DISCUSSION

We conducted a prospective cohort study of metagenomic, metabolomic, and lipidomic associations with ICI response among NSCLC patients. We found that those with higher alpha diversity were somewhat more likely to have clinical benefit, and that there were differences in overall microbiome composition. We also found that higher relative abundances of *Bifidobacterium* and *Bifidobacterium longum* were associated with lower odds of clinical benefit. In contrast, we also found that those with a higher prevalence of *Ruminococcus* were more likely to have clinical benefit. Despite not quite reaching statistical significance after the Bonferroni correction, several metabolites and lipids were associated with clinical benefit highlighting the need to study these findings in larger populations. These included, 4-imidazoleacetate, involved in oxidation and the production of histamine^27^, and 6-bromotryptophan, a tryptophan derivative linked to lowering chronic kidney disease^28^, and some triacylglycerols involved in lipid and immune signaling.^29–31^ Some of these were also correlated with gut bacteria, suggesting potential interrelationships among the three markers as they relate to influencing ICI response.

We found that alpha and beta diversity were associated with clinical benefit. Many other studies have found similar results using fecal samples from NSCLC patients, as assessed via 16S rRNA gene sequencing. For example, Jin and colleagues in China found a strong positive association between alpha, beta diversity, and response to anti–PD-1 immunotherapy among 37 patients with advanced NSCLC (Shannon index, *p*=0.008).^26^ Zhang and colleagues found that higher alpha diversity was associated with clinical benefit among 70 patients with advanced NSCLC, also in China, while no differences in beta diversity were observed.^32^ In our study, an increase in alpha diversity associations was similarly associated with higher odds of clinical benefit, although these associations did not reach statistical significance, likely due to sample size. Beta diversity was statistically significantly associated with clinical benefit, which is consistent with recent findings among NSCLC patients.^33–35^

Our findings suggested that higher abundances of the genus *Bifidobacterium* and the species *B. longum* were associated with lower odds of clinical benefit. *B. longum* is known to exhibit anti-inflammatory properties,^36^ and abundance of *B. longum* has previously been associated with higher likelihood of anti-PD-L1 therapy response among patients with NSCLC,^26^ in addition to other cancers (melanoma^9^ and colorectal cancer^37^). In contrast, *Bifidobacterium* has been associated with invasive potential in immunocompromised hosts, contributing to a sepsis-like presentation.^38^ We also found the presence of the genus *Ruminococcus* in NSCLC patients was associated with higher odds of clinical benefit. Prior literature has reported mixed findings across the different taxonomic levels. In contrast to our findings, *Ruminococcus species*, such as *R. bromii* and *R. unclassified*, were previously found to be less prevalent among NSCLC patients who had clinical benefit from ICI.^39^ Lower abundance of the *Ruminococcaceae* family has been reported among NSCLC patients who have controlled disease compared to those with disease progression.^40^ However numerous other recent studies showed positive results with Ruminococcus, both in animal models such as mice with enriched gut *Ruminococcus gnavus*,^41^ and human studies,^42–44^ which demonstrated enhanced anti-PD-1 tumor immunotherapy efficacy with Ruminococcus enriched in the microbiome of lung cancer patients. This positive effect in lung cancer and other solid tumors treated with immunotherapy may be due in part by its positive effects of decreasing intestinal permeability and its role in generating short chain fatty acids such as acetate and propionate. Taken together, our findings reflected the remarkably inconsistent nature of the microbiome-ICI response literature, as described below.

That there are some inconsistencies such that some bacteria have been associated with ICI response in some studies while in other studies they have the opposite association is well described.^45,46^ Possible explanations for our contradictory finding include an inability to resolve strain-level differences which may contribute to variation in species-level associations. For example, genomic analyses have shown functional diversity among *B. longum* subspecies, and some current taxonomic classification methods may only capture a subset of strains and their functional capacity.^47,48^ However, it is well described that distinct microbiome-ICI response associations can be observed across different geographic locations/ethnic/population characteristics.^49,50^ Finally, bacteria have remarkable *multifunctionality* that is enhanced with higher diversity.^51^ This functional redundancy likely explains why different specific bacterial taxa in different microbial environments, in response to the presence of differing bacteria, may indicate either positive or negative associations with immunotherapy responses.

Inconsistencies in the microbiome-ICI findings may be resolved to some extent by studying the metabolites produced and regulated by gut microbes. In our study, the serum metabolites 4-imidazoleacetate and 6-bromotryptophan, as well as some plasma triacylglycerols, were associated with higher likelihood of ICI-clinical benefit; however, these associations did not quite reach statistical significance after correction for multiple comparisons. Imidazole-containing compounds have been extensively studied due to their antibacterial, antifungal, antihistamine, anti-inflammatory, and anticancer properties.^52–54^ Furthermore, 6-bromotryptophan is a derived metabolite of tryptophan metabolism and a post-translational protein modification specific to the *E. coli K-12* strain MG1655,^55^ and has been associated with a lower risk of chronic kidney disease progression.^28,56^ Cholesterol cellular metabolism, such as that of triacylglycerols, has also been linked to lung cancer development and progression.^29^ Several metabolites and lipids were correlated with bacterial species elucidating the potential links between the different physiologic pathways.

### LIMITATIONS

There were study limitations such as the variation in the microbiome-ICI associations by participant characteristics; however, our sample size was too small for stratified analysis. Our study also had several strengths. We used a detailed multi-omic approach to characterize the response to ICIs and were able to characterize covariates in detail to account for potential confounding.

## CONCLUSIONS

The somewhat positive association between alpha diversity and clinical benefit reinforces the idea that a more diverse microbiome is healthier and predisposes patients for a better immunotherapy response. Taken together, we found that the gut microbiome and related metabolites and lipids may be associated with ICI clinical benefit among NSCLC patients. We found that multiple bacteria including the prevalence of *Ruminococcus*, and the abundance of *Bifidobacterium species* exhibited opposing associations with clinical benefit in NSCLC patients. Notably, many of our microbiome findings conflicted with some other microbiome-ICI studies, a contradiction which has been well described previously.^51^ We also found that metabolites, including 4-imidazoleacetate and 6-bromotryptophan, and lipids had uncorrected associations with ICI clinical benefit, possibly due to their inhibition of lung cancer cell proliferation. Clinical benefit trended toward an association with glucose and/or fructose, indicating a complex interplay between microbiome, lifestyle, and clinical benefit. This highlights a need to resolve these associations using a detailed multi-omic approach in larger study populations that have sufficient sample size for population characteristic stratification.

## Supplementary Material

Supplementary Files

This is a list of supplementary files associated with this preprint. Click to download.

- SupplementaryTable1.docx
- SupplementaryTable2..docx
- SupplementaryTable3.docx
- SupplementaryTable4.docx
- SupplementaryTable5.docx
- SupplementaryTable6.docx
- SupplementaryTable7.docx
- SupplementaryFigure1.jpeg
- SupplementaryFigure2.jpeg

## Figures and Tables

**Table 1:** Demographic and clinical characteristics for a cohort of non-small cell lung cancer patients (N = 66)

Patient Characteristics	No Clinical Benefit, N = 32 (%)	Clinical Benefit, N = 34 (%)	P-value^1^
Age at Diagnosis, years, mean ± SD	64.8 **±** 8.2	66.0 **±** 9.5	0.57
Gender			0.82
Female	16 (50.0)	19 (55.9)	
Male	16 (50.0)	15 (44.1)	
Race			0.7
White or Caucasian	27 (84.4)	31 (91.2)	
Black or African American	3 (9.4)	2 (5.9)	
Asian Indian, Pakistani	1 (3.1)	0 (0.0)	
Other	1 (3.1)	1 (2.9)	
BMI, kg/m^2^, mean ± SD	25.7 **±** 7.6	26.1 **±** 4.8	0.82
% Ideal Body Weight **± SD**	122.8 **±** 39.5	125.8 **±** 23.8	0.71
Diabetes, yes	9 (28.1)	4 (11.8)	0.17
Charlson Comorbidity Index Score, mean ± SD	6.1 **±** 2.33	6.4 **±** 2.6	0.64
Smoking Status			0.76
Current	11 (34.4)	9 (26.5)	
Former	17 (53.1)	21 (61.8)	
Never-smoker	4 (12.5)	4 (11.8)	
Pack-years (current or former smokers), mean ± SD	26.0 **±** 27.7	31.5 **±** 24.2	0.39
Special Diet			0.22
No	29 (90.6)	34 (100.0)	
Pescatarian	3 (9.4)	0 (0.0)	
Pre-treatment Antibiotic Use, yes	16 (50.0)	21 (61.8)	0.46
Pre-treatment Pro/Prebiotic Use, yes	4 (12.5)	3 (8.8)	0.93
Pre-treatment Steroid Use, yes	15 (46.9)	24 (70.6)	0.09
Pre-treatment GI Medication Use, yes	15 (46.9)	24 (70.6)	0.66
Stage			1.00
IIIA or IIIB	2 (6.2)	2 (5.9)	
IV	30 (93.8)	32 (94.1)	
Brain metastasis	7 (21.9)	9 (26.5)	0.73
Cancer Cell Type			0.46
Adenocarcinoma	21 (65.6)	19 (52.9)	
Squamous Cell Carcinoma	4 (12.5)	4 (55.9)	
Other^2^	7 (21.9)	11 (32.4)	
PDL-1 Result			0.33
Positive	15 (46.9)	20 (58.8)	
Negative	13 (40.6)	8 (23.5)	
Not tested	4 (12.5)	6 (17.6)	
PDL-1, if positive %, mean ± SD	52.3 **±** 32.8	54.2 **±** 35.5	0.88
Received Immunotherapy plus Chemotherapy, yes	21 (65.6)	22 (64.7)	0.72
Received Immunotherapy plus Targeted Therapy, yes	11 (34.3)	12 (35.3)	0.85
ECOG Performance Status			0.57
Fully active	4 (12.5)	5 (14.7)	
Symptomatic but completely ambulatory	1 (3.1)	0 (0.0)	
**Progression-Free Survival, days, mean ± SD**	**115.59 ± 85.75**	**434.18± 173.27**	**<0.001**

1 P-values were calculated using chi-squared test for categorical variables, ANOVA for normally distributed continuous variables, and Kruskal-Wallis test for non-normally distributed continuous variables.

2 Other cell types include non-small cell lung carcinoma NOS, adenosquamous carcinoma, and acinar cell carcinoma.

## Data Availability

The whole genome sequencing data generated for this study are publicly available in the International Nucleotide Sequence Collaboration (INSDC) repository http://www.ncbi.nlm.nih.gov/bioproject/1455662. Due to the sensitivity of patient confidentiality, the authors can make access to the clinical data available upon request, subject to approval from the institutional review board.
